# Toward the establishment of best practice guidelines for human research in Genomic Medicine in Brazil

**DOI:** 10.1590/1678-4685-GMB-2024-0230

**Published:** 2026-03-16

**Authors:** Fernanda Sperb Ludwig, Osvaldo Artigalás, Nureyev Ferreira Rodrigues, Patricia Ashton Prolla, Ursula da Silveira Matte, Jonas Alex Morales Saute

**Affiliations:** 1 Hospital de Clínicas de Porto Alegre, Diretoria de Pesquisa, Porto Alegre, RS, Brazil.; 2 Hospital de Clínicas de Porto Alegre, Centro de Pesquisa Experimental, Basic Research and Advanced Insvestigation in Neuroscience (Brain), Porto Alegre, RS, Brazil.; 3 Universidade Federal do Rio Grande do Sul, Programa de Pós-Graduação em Genética e Biologia Molecular, Porto Alegre, RS, Brazil.; 4 Universidade Federal do Rio Grande do Sul, Departamento de Genética, Porto Alegre, RS, Brazil.; 5 Hospital de Clínicas de Porto Alegre, Programa de Medicina Genômica e de Precisão, Porto Alegre, RS, Brazil.; 6 Hospital de Clínicas de Porto Alegre, Centro de Pesquisa Experimental, Laboratório de Medicina Genômica, Porto Alegre, RS, Brazil.; 7 Universidade Federal do Rio Grande do Sul, Programa de Pós-Graduação em Medicina: Ciências Médicas, Porto Alegre, RS, Brazil.; 8 Universidade Federal do Rio Grande do Sul, Faculdade de Medicina, Departamento de Medicina Interna, Porto Alegre, RS, Brazil.

**Keywords:** Human genomics, medical genomics, variant classification, genetic counseling, genomic data

## Abstract

Many countries lack their guidelines for best practices in genomic medicine, whether for clinical practice or research. For the construction of these national guidelines or institutional policies in human genomics research several key criteria must be observed. The determination of variant pathogenicity has become more refined, with specific global recommendations being adopted. A well-conducted consent process, managed by qualified professionals, ensures the protection of research participants’ rights. Pre-established sequencing and data analysis parameters are crucial for ensuring reliable results, and clear guidelines must be provided on whether or not to disclose findings to participants. Data storage remains a challenge due to both the size and sensitivity of the generated files. This work discusses these critical aspects and represents an initiative to propose the best practice recommendations for genomic medicine in human research. These recommendations were developed by a working group of experts and were further refined through discussions with members of the scientific community in genetics and genomic medicine.

## Introduction

Advances in genomic medicine for diagnostic purposes are among the most dynamic and promising areas of medicine and are poised to revolutionize the understanding of human health. Research in human genomics has grown exponentially in the past decade, resulting in enhanced diagnostic, prognostic, and treatment protocols, facilitating the identification of new pathologies and enabling genetic counseling for families. Genomic medicine programs have been established globally in public and private institutions, with their potential to revolutionize medical practice already evident in the daily lives of professionals and patients. However, many countries still lack their guidelines for best practices in genomic medicine, whether in clinical practice or research. In the United States and Europe, various thematic groups engage in discussions on the subject and disseminate their findings through documents and scientific articles to guide the field. These groups aim to represent public and private institutions, genetic service providers, and patients by offering education and resources for genetics and genomics practice. They develop guidelines and educational materials for diagnostic purposes, which are often adopted by countries without their own legislation or recommendations. The prominent groups in this area are the American College of Medical Genetics and Genomics (ACMG), Association for Molecular Pathology (AMP) and ClinGen - The Clinical Genome Resource, which serves as a key source of information. Additionally, select European, American, and Canadian groups are part of this network (Richards et al., 2008; Rehm et al., 2015; Ginsburg et al., 2021; Kumar, 2025).

The availability of diagnostic tests for patient care has been established in Brazil for a few years, particularly in the field of genomics. However, this remains a relatively new area for many healthcare professionals responsible for requesting these tests. There is a growing demand for professionals proficient in conducting these tests and for educational initiatives to train them. These efforts aim to increase awareness about selecting the most appropriate tests and interpreting the results. Such tests give patients greater access to health information (Kurnat-Thoma, 2020). Additionally, genomic tests involve handling sensitive information and raising ethical, social, and legal issues related to diversity, equity, and individual inclusion (Green et al., 2020; Miller et al., 2021).

Brazil has legislation to control human research through Law No. 14,874/2024, which regulates research on human beings and establishes the National System of Ethics in Research, with local research ethics committees (CEP) and the National Commission for Ethics in Research (CONEP). Regulations on genetic studies are governed by the National Biosafety Council (CNBS) and the National Technical Commission for Biosafety (CTNBio, 2024), which establishes the National Biosafety Policy (PNB). There is no specific regulation for genomic studies. Studies conducted on humans must be approved by the local or even national research ethics committee. However, when the research topic involves genomic analysis, there are no references or legal basis for conducting decisions (CTNBio, 2024; Ministério da Saúde, 2024). 

This paper presents one of the first reports of a Brazilian initiative toward the establishment of best practices in genomic medicine research through a guiding document. It provides recommendations for conducting projects involving human genomic analysis of germline alterations. The document was developed by a working group of experts in medical genetics, molecular biology, and bioinformatics between 2023 and 2024 in 10 synchronous meetings. The final version of the document was exposed to the group of genomic medicine implementation project at the Hospital de Clínicas de Porto Alegre (HCPA - FIPE 2021-0418) with 12 researchers in the field of genomics and subsequently underwent public consultation among the community of 4,195 professionals at the HCPA through a Google Forms with an open field for questions and suggestions.

## Good practices in Genomic Medicine studies 

Genomic Medicine involves diagnosing, predicting, prognosticating, preventing, or treating diseases using informed approaches enabled by knowledge of the genome and its encoded molecules. Its applications span research and interventions for rare and common complex diseases (Olorunsogo et al., 2024).

This document outlines key considerations for planning and conducting research in Genomic Medicine, specifically those involving genomic analyses in humans that could impact individuals tested. For the purposes of this document, “genomic tests” refer to those evaluating the entire genome, exome, or extensive gene panels, not solely genes associated with the condition under investigation. We recommend adherence to established guidelines, such as those from ACMG/AMP and Clingen, and further discuss their application in the context of human research (Richards et al., 2008; Rehm et al., 2015). It is important to recognize that multiple local guidelines may diverge from ACMG/AMP standards, and heterogeneity in variant classification and result interpretation remains a major challenge for the effective and globally coherent use of genomic data. The ACMG/AMP guidelines, along with ClinGen working groups responsible for their refinement and harmonization, represent the most widely adopted international standards. In cases of disagreement regarding their application in specific contexts, collaborative mechanisms are available to support the ongoing revision of these documents - eliminating the need for parallel initiatives, which often reproduce the same challenges that prompted the creation of efforts like ClinGen. Although ClinGen is funded by the NIH in the United States, it includes contributors from 72 countries, underscoring its global scope. Nonetheless, local implementation of these guidelines may vary considerably, reinforcing the importance of developing national or regional guides for use. A summary of the ACMG/AMP and Clingen recommendations is available in Table 1 and a glossary of terms used in genomics is available in Table 2.

**Table 1 -:** Summary of the recommendations.

Action	Recomendation
Good practice	Follow ACMG recommendations and Clingen updates, as well as instructions of regulatory groups and legislation in your country
Primary findings	Report variants P or LP associated with the trait under investigation Report VUS in genes relevant to the phenotype under investigation when no P or LP are identified Emphase the VUS potential for reclassification over time Do not report B and LB variants.
Secundary findings	Being the option of the research group to report SF, is suggested offering patients the option to receive results related to actionable genes in the ICF during pre-test counseling To check updated versions of the ClinGen working group’s recommendations for SF In children and adolescents include benefits that could directly affect their age group or the child’s parents
Genetic Counseling	Must be conducted by a qualified professional In pre-test is crucial to emphasize the scope and capability of the test In post-test should deliver and explain the results obtained
Consent process	All research participants must sign an ICF or Free and Informed Assent Form (FAF) with the option to decline: Detail in the ICF the type of genomic test performed and the potential implications of the results If applicable, information must be included about access to raw genomic data, timeframe for accessing, authorization for depositing data in an institutional database, following an opt-out approach
Reports	Should include, if applicable: Primary findings and interpretation Findings on actionable genes and interpretation Description of sequencing methods and quality parameters
Pseudonymization and Anonymization of Data	Use anonymization or pseudonymization codes as a standard for identifying files containing raw and/or processed genetic data Implement security measures to prevent unauthorized access Comply the country data protection law
Data Storage	Taking into account factors such as research objectives, associated costs, and reasons for data retention. It is recommended for research projects deposit resulting data into an institutional database.
Public Database	It is recommended, as a best practice in research, to deposit genomic data generated in public repositories Consent indications from participants and data anonymization must be considered
Collaborative International Studies	Researchers must adhere to the recommendations and regulations of the national research ethics commission of the national health council in all instances, as well as comply with the general data protection laws

Legend: P: Pathogenic; LP: Likely Pathogenic, B: Benign, LB Likely Benign, SF: secondary findings

**Table 2 -:** Glossary of genomic terms.

Term	Definition
Genomic tests	These evaluate the entire genome, exome, or broad gene panels, not limited to genes related to the condition investigated
Primary findings	Variants, known or uncertain, in genes related to the investigated condition, whether pathogenic or not
Secondary findings (incidental findings)	Variants, known or uncertain, in genes unrelated to the condition investigated
Actionable findings	A type of secondary finding comprising variants of known significance in genes associated with diseases where intervention can alter their natural course
Analysis	Process of examining an individual’s genomic data after testing
Reanalysis	Reviewing genomic data without repeating testing
Phenotyping	Collecting phenotypic information from medical records
Rephenotyping	Gathering additional phenotypic data through further examination of medical records
Alignment	Bioinformatics tools aligning sequence reads to a reference genome
Realignment	Realigning sequence reads, possibly to a newer reference genome version
Variant identification	Identifying genomic positions differing from the reference
Variant annotation	Describing a variant’s effect, from its position to protein/transcript impact
Prioritization	Assigning clinical/molecular significance based on variant-gene-disease associations and population database allele frequencies
Reprioritization	Reevaluating variant significance with updated gene-disease relationship information
Assessment	Detailed variant analysis considering allele frequencies, segregation, and functional/computational data, following established criteria
Variant classification	Determining variant pathogenicity using guidelines (e.g., ACMG criteria)
Report issuance	Producing a clinical report summarizing analysis results
Genomic counseling	Counseling patients on genomic testing and results
Reuse	Utilizing existing genomic information in other healthcare contexts
Expanded genetic testing	Using additional tests to gather more DNA sequence data, including methodologies like exome sequencing or orthogonal methods such as microarrays
Data Sharing	Sharing individual information, variant curation results, etc., in publicly accessible databases

One key aspect of genomic analyses, unlike other tests, is its ability to yield information on a large number of genes, many unrelated to the specific trait being studied. This information may have implications either in the participant’s future health or in clinical applications whose utility remains uncertain. Germline and somatic genomic testing in clinical and research settings can reveal primary and secondary (or incidental) findings. There is no consensus on whether findings from research genomic tests, including secondary ones in actionable genes, should be reported. Therefore, researchers must decide beforehand, considering the risks and benefits involved, and clearly communicate this decision in the Informed Consent Form (ICF), detailing potential repercussions in each scenario.

Hence, the importance of pre-test counseling is emphasized to establish clear expectations regarding which types of results will be reported and explained. Researchers in genomic medicine must ensure participants have access to professionals experienced in genetic counseling.

The research project and ICF must specify what information will be disclosed to participants upon completion of testing, including whether a research report will be provided and the nature of its contents. During genetic counseling, the physiological basis of the disease, its pattern of expression, and its penetrance must be clearly communicated to the research participant. Further recommendations regarding the report and genetic counseling process are outlined below.

We endorse the guidelines of the ACMG/AMP as best practice (https://www.ncbi.nlm.nih.gov/clinvar/docs/acmg/) and recommend adopting similar standards, as well as checking the available literature according to the chosen sequencing method or platform and parameters. Different methods of sequencing must be evaluated, library construction based on amplification, capture, or even new technologies such as CRISPR-based and adaptive sampling are options, and all have different advantages and disadvantages (Richards et al., 2008; Fang et al., 2014; Wang et al., 2017; Barbitoff *et al.*, 2020; Przybyla and Gilbert, 2022). 

Regarding sequencing, the following minimum coverage parameters are recommended for utilizing sequencing data:

Germline gene panels: average coverage of target regions (coding regions and splice sites of analyzed genes >150x, with at least 97% of these regions having coverage greater than or equal to 50x. Check at least the CDS region of the top transcript and the pattern of disease-causing splice site variants for the disease group you work with. Most splice site variants involve primarily five nucleotides, and there are exceptions with known variants deeper in the intron (Scotti and Swanson, 2016). 

Exomes: average coverage of target regions (coding regions and splice sites of analyzed genes) >50x, with at least 97% of these regions having coverage greater than or equal to 20x.

Whole genomes: average coverage of target regions >30x, with at least 97% of these regions having coverage greater than or equal to 10x.

A Phred score of Q30 or higher should be sought for base quality for all types of genomic tests mentioned above. Phred scores lower than 30 can lead to false positives as bases are called incorrectly, and high thresholds can exclude correct bases, resulting in a loss of valuable information (Liao et al., 2017). 

### Primary findings

Primary findings are variants in genes associated with the trait under investigation, whether pathogenic or not. This concept applies exclusively to traits with a known genetic basis and a monogenic or oligogenic inheritance pattern.

The results presented in the research report should be clarified during post-test counseling. In such cases, we recommend reporting pathogenic or likely pathogenic variants and considering whether to include the broader implications of these findings. Broad implications refer to other traits influenced by the same gene or variant but not directly related to the trait under investigation. The decision to report broader implications should mirror the considerations for actionable findings (see below).

Suppose the researcher opts to disclose broader implications. In that case, the following phrasing is suggested: “This variant is associated with the studied condition and has also been reported in another condition (describe), which currently does not manifest in the participant.”

We suggest reporting variants of uncertain significance (VUS) in genes relevant to the phenotype under investigation, particularly when no pathogenic or likely pathogenic variants are identified. Whenever VUS is reported, the classification details should be provided, emphasizing its potential for reclassification over time. We advise against reporting benign and likely benign variants in the research report to minimize confusion.

### Secondary findings

Secondary findings, also known as incidental findings, are variants in genes unrelated to the condition under investigation. Generally, these findings should not be disclosed unless they fall under actionable gene findings.

Actionable gene findings involve pathogenic or likely pathogenic variants in genes associated with diseases for which interventions can alter their natural course. Despite various guidelines recommending different approaches, consensus on whether to report actionable gene findings remains elusive. However, widely recognized international guidelines, such as those from ACMG/AMP, recommend that patients be given the option to receive results related to actionable genes. Updated versions of the ClinGen working group’s recommendations for secondary findings should be followed (https://www.clinicalgenome.org/working-groups/actionability/). Patient preference regarding the receipt of such results should be obtained during pre-test genetic counseling and documented in the ICF. 

Recent data from Brazil indicate that approximately 2.6% of exome sequencing cases in the context of rare diseases reveal secondary findings in actionable genes (Quaio et al., 2022). Recent data from Brazil indicate that approximately 2.6% of exome sequencing cases in the context of rare diseases reveal secondary findings in actionable genes (Quaio *et al.*, 2022). We emphasize the need for studies in the Brazilian population to propose additions or refinements to the ACMG/AMP list, based on local allele frequencies, disease penetrance, and the actual actionability of findings in our setting. It is important to note that the ACMG/AMP list uses FDA-approved therapies as one of its criteria, which may not always align with treatment approvals by the Brazilian regulatory agency (ANVISA, 2020). Therefore, national consensus efforts are needed to develop a context-specific list of actionable genes that reflects local cultural, regulatory, and technological realities. Until such local adaptations are established and broadly adopted, we recommend that the ACMG/AMP actionable gene list be considered the minimum standard for genomic analysis and reporting. We advise that participants should be informed during pre-test counseling about the possibility and implications of receiving secondary findings related to actionable genes listed by ACMG/AMP: https://www.ncbi.nlm.nih.gov/clinvar/docs/acmg/. If participants choose to receive these results, they should be included in a separate report section from the primary findings and explained during post-test counseling. Variants of uncertain significance (VUS), benign, and likely benign findings should not be reported for secondary findings. It is important to note that ACMG’s list of actionable genes is periodically updated, and the classification of VUS can change over time. Research groups should outline how they will handle updates to gene lists or variant classifications as actionable.

In the case of children and adolescents, considerations must be made regarding their limited autonomy and ability to comprehend the implications of genetic findings. Some findings may only impact the individual during adulthood. Despite these factors, arguments favoring reporting actionable gene findings in children and adolescents include benefits that could directly affect their health outcomes within their age group (Hunter et al., 2022). Additionally, early diagnosis and treatment options could benefit the child’s parents. There is no evidence of negative clinical consequences for participants in such cases, except for potential psychological impacts, which may vary based on the participant’s health status (e.g., symptomatic versus healthy controls) (Houdayer et al., 2019). The research team should carefully assess these factors when offering the option of receiving actionable gene findings, which must be a selectable option by legal guardians in the ICF (see the informed consent section below).

Genetic counseling

In the context of genomic research testing, both pre-test and post-test genetic counseling involve explaining the test’s nature and limitations, potential results, and implications for participants and their families, considering primary findings and findings on actionable genes. It is recommended that genetic counseling should be conducted by qualified professionals in accordance with professional councils. 

During pre-test counseling, it is crucial to emphasize the scope of the test - whether it covers the entire genome, exome, or specific gene panels. The test’s diagnostic capability for the specific trait should be explained; for instance, tests may provide diagnostic possibilities for monogenic or oligogenic diseases but may not contribute to the diagnosis of other conditions. The likelihood of obtaining positive or uncertain results, including findings on actionable genes, should be presented, along with their clinical and ethical implications for the participant and their family. Clear communication of the test’s limitations to the participant is also essential.

Post-test counseling should deliver and explain the results obtained. In cases where positive results arise, whether from primary findings or actionable genes, the implications of these results and potential therapeutic options should be discussed. The research team should guide participants in accessing appropriate healthcare services based on the needs identified through genomic testing.

## Consent process

All research participants must sign an ICF or Free and Informed Assent Form (FAF) in the case of children and adolescents. The ICF should detail the type of genomic test performed (panel, exome, or whole genome) and the potential implications of the results. It is suggested that the ICF default on reporting findings on actionable genes, with participants given the option to decline (opt-out). The participant’s decision must be clearly documented in the ICF or FAF. Additionally, the forms should specify whether participants will have access to raw genomic data and, if so, the timeframe for accessing this data. Authorization for depositing data in an institutional database should also be sought where applicable, following an opt-out approach.

### Reports

For research involving the analysis of traits with a known genetic basis and a monogenic or oligogenic inheritance pattern, a clinical laboratory research report is recommended. This report should include the following information:

Primary findings (main results) including gene name, genomic variant position, gene, zygosity, protein position, ACMG/AMP classification, and OMIM identifier. Genomic positions should adhere to Human Genome Variation Society (HGVS) guidelines (https://varnomen.hgvs.org/). If no causal variant is identified, this absence should be noted.

Interpretation of results, providing main clinical information related to the primary findings and details of the variants.

Results of findings on actionable genes follow the same recommendations as primary findings. If no pathogenic or likely pathogenic variants are found in actionable genes, indicate the absence of such variants in the analyzed genes.

Interpretation of results for findings on actionable genes, detailing the variants identified and their clinical implications.

Description of sequencing methods and quality parameters, including analysis platform, number of reads in variant regions, average coverage and depth, reference genome used, and methodology limitations. If orthogonal tests were used to confirm variants, these methods and results should be included in their respective sections. If the research group implements new techniques, it is recommended that orthogonal tests be carried out to validate the new method.

A clinical-laboratory research report should still be provided if participants consent to reporting findings on actionable genes for research not involving traits with a known genetic basis and a monogenic or oligogenic inheritance pattern (e.g., multifactorial or nondiagnostic characteristics). This report should include:

Results of findings on actionable genes, including gene name, genomic variant position, gene, zygosity, protein position, ACMG/AMP classification, and OMIM identifier. Genomic positions should adhere to HGVS guidelines. If no pathogenic or likely pathogenic variants are found in actionable genes, indicate their absence.

Interpretation of results for findings on actionable genes, detailing the variants identified and their clinical implications.

Description of sequencing methods and quality parameters, including analysis platform, number of reads in variant regions, average coverage and depth, reference genome used, and methodology limitations. If orthogonal tests were used to confirm variants, these methods and results should be included in their respective sections. Research reports must value analytical quality metrics. For commercial laboratories that provide diagnostic services, there is an ANVISA Laboratory Accreditation process, which aims to guarantee the quality, safety, and reliability of the services offered by clinical analysis laboratories, where laboratories undertake to follow specific standards and guidelines, aiming at the continuous improvement of processes and ensuring the accuracy of the results of the tests performed. Collegiate Board Resolution (RDC) 390/2020 can be consulted, which establishes rules for the operation of analytical laboratories, the procedures for their qualification in the Brazilian Network of Analytical Laboratories in Health (Reblas) and their accreditation to carry out orientation, control and fiscal analyses on products subject to health surveillance (ANVISA). The main recommendations in analytical laboratories are: 1) Documentation: records of experiments, documentation of all materials used including source, lot numbers, dates and tracking for electronic and physical storage; 2) Standardized Procedures: standard operating procedures (SOPs) for all laboratory activities; validation of all analytical methods in accuracy and reliability; trained team; 3) Quality Control: quality control program that includes regular checks on reagents, equipment, procedures and accuracy of measurements; control of environmental conditions, equipment maintenance and calibration; 4) Data Management: procedures for data collection, storage, analysis and accessibility; 5) Ethical Considerations: ensure privacy and confidentiality, follow ethical guidelines for research and institutional approval.

## Pseudonymization and anonymization of data

Data protection laws mandate the anonymization or pseudonymization of genomic data, which is considered sensitive due to its potential to identify participants. It is recommended that pseudonymization codes be used as a standard for identifying files containing raw and/or processed genetic data, coupled with implementing security measures to prevent unauthorized access.

Anonymization involves data processing techniques that prevent direct or indirect association with the participant’s identity. When anonymization is not feasible, pseudonymization techniques must be employed to comply with the country or regional data protection law. In Brazil, the law n°13,709 of August 14, 2018, governs data protection (https://www.planalto.gov.br/ccivil_03/_ato2015-2018/2018/lei/l13709.htm). Pseudonymization is a data processing method that involves using additional information (artificial identifiers) accessible only to data controllers (researchers), linked with information that could potentially identify the participant in which the key code is retained but kept separately, enhancing privacy-protecting measures. Pseudonymized data and anonymized data may also be treated differently in particular regulatory contexts. Participants must be informed in the consent form about how their data will be processed - anonymization or pseudonymization (O’Doherty et al., 2021; Wan et al., 2022).

Data storage

The research project should outline the storage of generated genomic data, taking into account factors such as research objectives, associated costs, and reasons for retention. It is recommended that genomic analysis research projects deposit resulting data into an institutional database, adhering to specified criteria.

Regarding data formats, it is advised to store genomic data in formats conducive to reanalysis or future investigations while minimizing project overhead. There are no recommendations for the storage time and format of genomic research data files, and we understand that format and storage decisions must be cost-effective for the research group. Priority should be given to storing data, for example, in the most commonly used FASTQ and uBAM formats (for raw sequencing data) and VCF format (for variant analysis data), as these are standardized formats enabling interoperability across databases and analysis software. Additionally, these formats are more compact than other analysis-generated files, reducing storage costs (Table 3). It is important for researchers to be aware that file storage formats such as ORA, D4, and CRAM can be more efficient in storage and read formats.

**Table 3 -:** Comparative table of file types and disk space utilization.

Sample	FASTQ^1^	BAM	VCF^2^
Exome*	~20GB	~4GB	~25MB
Genome**	~100GB	~80GB	~125MB

*30X medium coverage; **20X medium coverage; ^1^estimated size, reads 101nt long, uncompressed file; ^2^estimated size, uncompressed file.

If only reprioritization and reclassification of variants are planned, storing the VCF file will suffice. However, if data reanalysis is intended-such as using a different reference genome, alignment tool, or adjusting quality parameters-raw files (ex: FASTQ or uBAM) are necessary.

For storage, either a cloud solution or a physical server can be used according to the legislation of each country. In both cases, access to data must be restricted, and solutions aligned with the prevailing data security standards at the project’s inception should be chosen, with periodic reviews during the storage period. Regular data backups are essential, with frequency and methods tailored to data volume and criticality for research. Backups can be stored in the cloud, on physical servers, or external hard drives, depending on which option best secures resources against loss and unauthorized access.

Regarding storage duration, if data reanalysis is anticipated, researchers should determine storage times empirically. For instance, recent studies suggest that reanalysis within 6 months of initial analysis does not yield significant additional insights. Studies involving minors should ensure sufficient storage time if participants are granted future data access. Usually, laboratories and analysis software that offer data storage keep them for a minimum period of 5 years, however, the British Society for Genetic Medicine (BSGM) recommends that this period be at least 30 years, given the usefulness of genetic data information (https://bsgm.org.uk/).

Participants’ right to access their data should be respected; researchers must specify in the consent form how this information will be provided upon request and for how long these data can be made available. All costs associated with these processes should be considered and detailed in the project budget.

Public databases

It is recommended, as a best practice in research, to deposit genomic data generated in public repositories such as SRA (Sequence Read Archive - the primary repository for massively parallel sequencing, https://www.ncbi.nlm.nih.gov/sra), BIPMED (a Brazilian database, https://bipmed.org/pages/upload.html), Lattes Data (https://lattesdata.cnpq.br/), ClinVar (the principal database for variant classification, https://www.ncbi.nlm.nih.gov/clinvar/), and others to share information within the scientific community. It is crucial to emphasize that this deposition is for information sharing and should not substitute the responsibility of the research group for data storage.

Large biobanks that store biological specimens, clinical data, and genomic data are revolutionizing research by setting a new standard for analysis and innovation in genomics and personalized medicine. Hundreds of publications have already been made through the use of biobanks, such as those of the UK and Estonia (Allen et al., 2024; Milani et al., 2024). Major global initiatives have demonstrated that collaborative studies through biobanks enrich and bring statistical power to studies (Zhou et al., 2022; Gallagher et al., 2024).

Consent indications from participants through a consent form must be considered, alongside recommendations for data anonymization. Before submission, it is essential to verify compatibility with each database’s specific policies and guidelines, as requirements regarding data format, quality, and documentation may vary. Ensuring data has been thoroughly checked and validated before submission is critical to prevent errors or inconsistencies, accompanied by comprehensive documentation or descriptions.

It should be noted that specific scientific journals, particularly those with open-access policies, may require original genomic databases to be deposited in public repositories prior to article submission.

Storing and sharing data in institutional databases, when available, is pivotal for research. These databases should facilitate centralized collection, storage, and management of data, enabling access and use for research purposes by diverse groups. Similar to public repositories, adherence to established standards for data quality and integrity is essential to ensure information reliability. Institutional databases should support the integration of research data with clinical information from participants/patients while maintaining cybersecurity measures, anonymization protocols, and data access controls to protect patient privacy and comply with health research regulations.

When data is deposited in an institutional database (as stipulated in informed consent forms as a selectable option by the patient or legal guardian), the institution assumes ownership and responsibilities, including evaluation of data adequacy criteria and establishment of storage parameters (location, file types, costs, and duration). It is important to highlight that while data deposition in institutional biobanks is essential, it does not absolve the research team of their responsibility for overall data storage management.

### Collaborative international studies

When sending data and biological samples to other countries, researchers must adhere to the recommendations and regulations of the National Research Ethics Commission of the National Health Council in all instances, as well as comply with the general data protection laws. Specific cooperation agreements for data transfer (data transfer agreements) must be signed between the participating institutions. The possibility of sharing information must be clearly described in the research project and in the ICF. If this possibility arises after project approval, an amendment must be submitted to the research ethics committee system to modify the project.

For research involving analysis of genetic material from original populations, current national legislation is mandatory (i.e., Brazilian Law No. 13,123/2015 and Decree No. 8,772/2016). These laws establish the requirement for prior registration with the National System for Management of Genetic Heritage and Associated Traditional Knowledge (SisGen) to obtain authorization for accessing genetic heritage and associated traditional knowledge.

## Discussion

There is an urgent need for countries without specific regulations and recommendations to explore the intersection of current data generation models in genomic health with clinical care and the ethical, legal, political, and social issues arising from this intersection. Genomics research is increasingly being conducted by professionals without genetics training, reflecting trends in clinical practice. Studies have shown a lack of consensus on the specific knowledge needed for professionals to engage in genomics-related activities. The understanding that genetics is crucial for long-term human health and involves a range of issues that have yet to be fully explored and discussed among Brazilian researchers was the driving force behind the development of guidelines for conducting genomic research in humans (Ormondroyd et al., 2022; Coleman et al., 2023).

The scientific advancements in current genomic research are leading to a growing body of knowledge that presents both opportunities and challenges. Healthcare professionals will need to become increasingly familiar with this information. Understanding genetic variants is expanding, leading to new diagnoses and treatments, facilitating the use of advanced genomic medicines, and enhancing public awareness of the advantages of genomic medicine (Johnson et al., 2020; Spicer and Papanikitas, 2021).

When considering the impact of a genomic test, an important concept that professionals and participants must grasp when conducting such investigations is the diagnostic yield, which can range from 3 to 70% depending on the condition under study. This diagnosis can result in changes that will affect clinical management, surveillance, counseling, and identifying risks for family members. However, one of the issues that requires attention and reflection is cases where there is no definitive conclusion from the generated data. In this regard, the classification of variants is an ever-evolving topic, with many variants still failing to determine pathogenicity. This fact is supported by the observation that the frequency of variants of uncertain significance ranges from 5 to 85% among genomic studies for diagnostic purposes and may have an even lower frequency of conclusive results depending on the ancestry of the individuals studied due to underrepresentation in databases. When investigating rare diseases, the evidence supporting variant classification is often limited, making it challenging to assign biological significance. The advancement of knowledge, continuous publication of research data, and the growth of population databases interpret genomic data as a dynamic process that can evolve, particularly in the case of variants of uncertain significance, which may benefit from revisiting and reanalyzing results (Graham et al., 2021; Shickh et al., 2021). 

Another driving factor behind the development of this document is the essential knowledge required to accurately identify relevant variants by defining and evaluating the test, considering factors such as coverage metrics, depth, and consistency of data to achieve the necessary quality for sound research. Professionals unfamiliar with genomic analyses need to prioritize data quality to produce reliable results. Understanding the scope and limitations of a genomic test requires technical knowledge that is crucial for selecting the appropriate test parameters. Differences in test selection- whether panels, exomes, or complete genomes- are essential for maximizing diagnostic efficiency based on the disease or trait under study (Koboldt, 2020; Marshall et al., 2020). 

One of the main challenges in analyzing genomic data across exomes and whole genomes is returning information to research participants. A plan is recommended to guide such a decision, regardless of the outcome, that is explicitly communicated to the participant. It is essential to consider the ability to communicate findings and manage them. When results are returned to research participants, weaknesses in the interpretation of results must be observed concerning the genetic determinism of a potential diagnosis, highlighting the importance of a trained team for communication, as results can be overestimated (Halverson et al., 2020; Graham et al., 2021).

The relationship with research participants should be founded on transparency and trust, while the collection and use of data should be questioned and presented clearly and objectively. Previous studies have shown that some participants either do not recall their consent decisions or remember them inaccurately. Data governance frameworks are still under development to regulate data sharing, management, and storage practices (Halverson et al., 2020; Juengst, 2021; Kabata and Thaldar, 2023; O’Doherty *et al.*, 2023).

The storage of participant information in institutional databases is crucial for the progress of genomics research. Still, debates arise around the obligation for participants to share their data in exchange for third-party information to interpret their results, as well as external funding to obtain the data. The integration of historically distinct research and clinical practices prompts ethical questions about the governance of such practices, while concerns about privacy and security challenge conventional notions of consent (Johnson et al., 2020; Ormondroyd et al., 2022; Lynch et al., 2023).

This document is expected to offer information and stimulate discussions for researchers, serving as a benchmark of best practices in conducting research. If not used in its entirety, it should highlight issues that deserve further consideration by researchers.

## Data Availability

Data can be shared upon request to the authors.
